# A G-Quadruplex Structure in the Promoter Region of *CLIC4* Functions as a Regulatory Element for Gene Expression

**DOI:** 10.3390/ijms19092678

**Published:** 2018-09-10

**Authors:** Mu-Ching Huang, I-Te Chu, Zi-Fu Wang, Steven Lin, Ta-Chau Chang, Chin-Tin Chen

**Affiliations:** 1 Department of Biochemical Science and Technology, National Taiwan University, Taipei 106, Taiwan; d02b22005@ntu.edu.tw; 2 Institute of Atomic and Molecular Sciences, Academia Sinica, Taipei 115, Taiwan; r04223204@ntu.edu.tw (I.-T.C.); f96223151@gmail.com (Z.-F.W.); tcchang@po.iams.sinica.edu.tw (T.-C.C.); 3 Institute of Biological Chemistry, Academia Sinica, Taipei 115, Taiwan; stevenlin@gate.sinica.edu.tw

**Keywords:** G-quadruplex, transcriptional regulation, promoter, CRISPR/Cas9

## Abstract

The differential transcriptional expression of *CLIC4* between tumor cells and the surrounding stroma during cancer progression has been suggested to have a tumor-promoting effect. However, little is known about the transcriptional regulation of *CLIC4*. To better understand how this gene is regulated, the promoter region of *CLIC4* was analyzed. We found that a high GC content near the transcriptional start site (TSS) might form an alternative G-quadruplex (G4) structure. Nuclear magnetic resonance spectroscopy (NMR) confirmed their formation in vitro. The reporter assay showed that one of the G4 structures exerted a regulatory role in gene transcription. When the G4-forming sequence was mutated to disrupt the G4 structure, the transcription activity dropped. To examine whether this G4 structure actually has an influence on gene transcription in the chromosome, we utilized the CRISPR/Cas9 system to edit the G4-forming sequence within the *CLIC4* promoter in the cell genome. The pop-in/pop-out strategy was adopted to isolate the precisely-edited A375 cell clone. In CRISPR-modified A375 cell clones whose G4 was disrupted, there was a decrease in the endogenous *CLIC4* messenger RNA (mRNA) expression level. In conclusion, we found that the G4 structure in the *CLIC4* promoter might play an important role in regulating the level of transcription.

## 1. Introduction

Chloride intracellular channel 4, CLIC4, is a multifunctional protein. In addition to its diverse physiological functions [1,2,3,4], the differential expression of CLIC4 between cancer cells and the surrounding stroma has been reported in various tumor types [5]. During cancer development, CLIC4 is downregulated in cancer cells, and it is recognized as a suppressor for tumor growth [6]. In the tumor stroma, on the other hand, CLIC4 is often upregulated and it plays a critical role in myofibroblast transition [7]. The opposite was found as early as at the transcription level [5], and both mechanisms could promote tumor progression. However, how CLIC4 is regulated remains unknown.

The regulation of gene expression includes multiple mechanisms such as transcription factor binding [8] and epigenetic modification, which involves DNA methylation, histone modification, and microRNA interaction [9]. Moreover, the DNA and messenger RNA (mRNA) sequences that would adopt alternative secondary structures have significant impacts on the transcription and translation of gene expression. Although CLIC4 has been identified for nearly 30 years [10,11], only NANOG, p53, c-Myc, and SP-1 transcription factors have been reported to regulate CLIC4 expression. Genome-scale location analysis revealed that NANOG and SOX2 were bound to CLIC4 early in embryonic stem cells [12]. CLIC4 was elevated in p53- or c-Myc-overexpressing cells that induced apoptosis with direct binding to the *CLIC4* promoter [13,14] and SP-1 was involved in Ca^2+^-induced keratinocyte differentiation [15]. Nevertheless, transcriptional regulation of the *CLIC4* promoter is still unclear.

Guanine-rich (G-rich) sequences containing four runs of G-tracts can adopt four-stranded structures, named the G-quadruplex (G4). Four guanine bases can assemble into a square planar structure via Hoogsteen-type hydrogen bonds, and this is called a G-quartet. A stack of G-quartets is stabilized by monovalent cations, such as potassium or sodium, and this forms a tetrahelical G4 structure. The loop sequences connecting G-tracts determine the types of G4 topology [16]. G4 architecture was first discovered in vitro more than 50 years ago [17]. The biological relevance of G4s then emerged since the late 1980s [18]. In the past few years, the existence of G4s in the human genome was directly visualized by using an antibody against the G4 structure [19], and fluorescent G4 probes [20].

G4s were found predominately in telomeres, and in the gene regulatory region of the human genome [21]. Particularly, a number of G4s have been recently found in eukaryotic promoter regions, including c-Myc, KRAS, PDGF, BCL-2, etc. [22,23,24,25,26]. According to current knowledge, G4 structures in promoter regions may influence transcription in both positive and negative ways. This also depends on the strand that G4 locates in, and the function of the proteins that are bound to the G4 structures. In addition, G4 formation could also affect protein binding. This is based on the function of the affected proteins; for example, whether the protein is a transcriptional activator or a repressor, and the proteins that stabilize or resolve the G4 structure, which could also result in different outcome [27]. Upon analyzing the *CLIC4* promoter, we found that high GC contents could form G4 structures. We further evaluated the effect of the G4 structure in regulating *CLIC4* gene expression.

## 2. Results

### 2.1. CLIC4 Promoter Analysis

**Figure 1:**
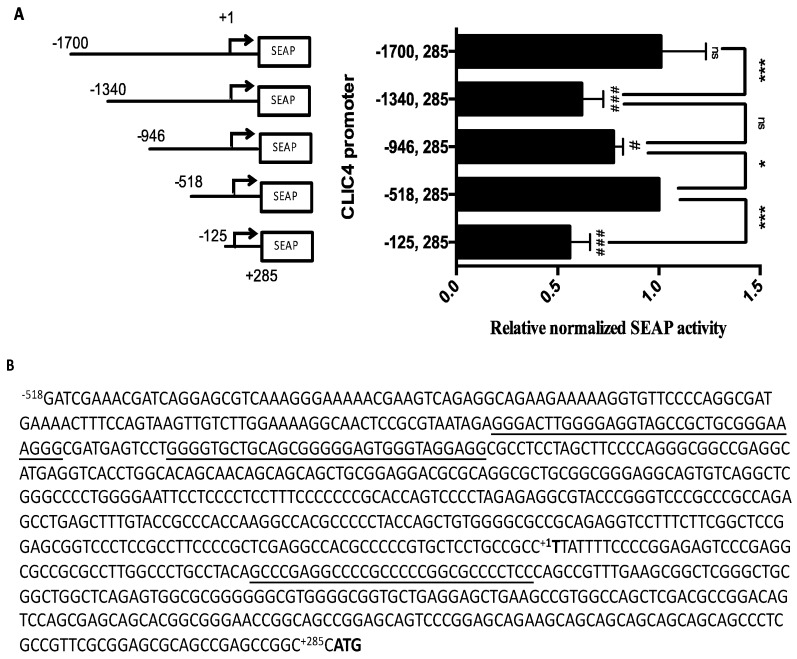
(**A**) A series of truncated *CLIC4* promoter regions were constructed in the pSEAP2-Basic vector, and the relative activities of the *CLIC4* promoter were compared to p(−518, +285). After transfection in A375 cells for 48 h, media were collected and a secreted embryonic alkaline phosphatase (SEAP) assay was performed. +1 indicates the transcription start site (TSS). Data are expressed as the means ± SD of three replicates. # *p* < 0.05, ### *p* < 0.001, as compared to p(−518, +285). * *p* < 0.05, *** *p* < 0.001 as compared to the adjacent promoter region. ns: non-significant difference; (**B**) Sequence of the *CLIC4* promoter (−518, +285). The underlined sequences are potential G-quadruplexes (PG4s).

To examine the regulatory region of the *CLIC4* promoter, a series of truncated *CLIC4* promoter sequences from −1700 to +285, relative to the transcription start site (TSS, +1) were cloned into the secreted embryonic alkaline phosphatase (SEAP) reporter vector pSEAP2-basic, and transfected into A375 cells to estimate the promoter activity of each region. As shown in Figure 1A, the longest construct p(−1700, +285) displayed the highest SEAP activity. The deletion of −1700 to −1340 at the promoter region resulted in a significant decrease in the SEAP activity. However, in p(−518, +285), the SEAP activity reversed to as high as p(−1700, +285), but further deletions of −518 to −125 resulted in a 50% drop in promoter activity. These results indicated that there are positive regulatory sites that are located in −1700 to −1340, and in −518 to −125, and negative regulatory sites that are located in the region between −1340 to −518.

During the reporter plasmid constructions with different promoter regions, we found that the PCR and sequencing reactions were hampered. Since high GC contents were found in the *CLIC4* promoter region, we argued that DNA secondary structure might exist to interfere with the PCR and the sequencing process. Therefore, this prompted us to analyze its promoter sequence with DNA secondary structure. The QGRS program [28] predicted that there was high G4 density in the region between −518 and +285. The promoter sequence of *CLIC4* p(−518, +285) is shown in Figure 1B.

### 2.2. Putative G-Quadruplexes in the *CLIC4* Promoter

**Figure 2:**
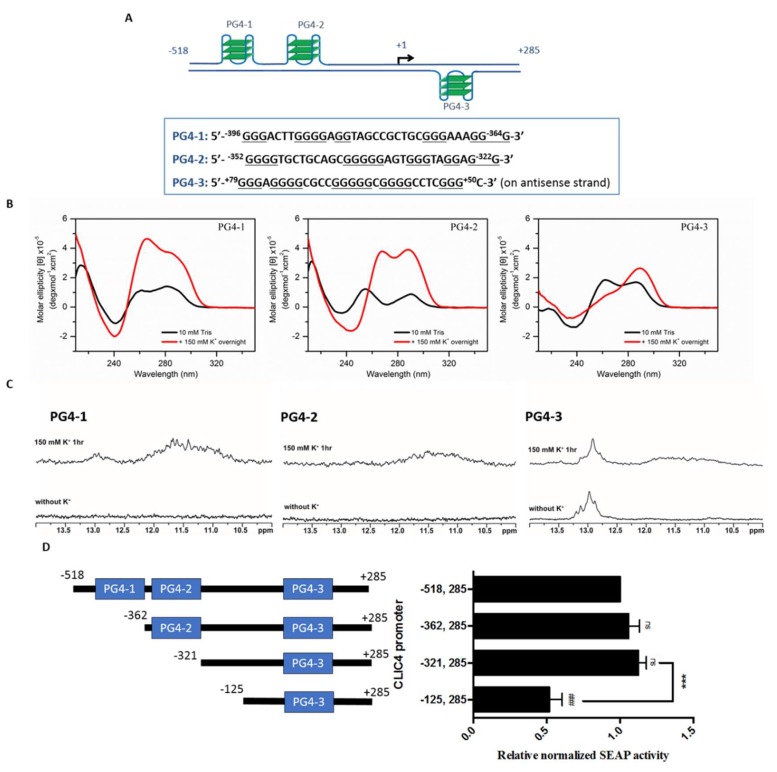
Putative G-quadruplexes (PG4s) in the *CLIC4* promoter (−518, +285) (**A**) Schematic representation of three PG4s and their sequences in *CLIC4* promoter. G-tracts that might participate in G4 formation were underlined; (**B**) The circular dichroism (CD) spectra of three PG4s in Tris-HCl buffer without (black line) and with (red line) 150 mM KCl for overnight; (**C**) The imino proton nuclear magnetic resonance spectroscopy (NMR) spectra of three PG4s in Tris-HCl buffer without (bottom panel) and with (top panel) 150 mM KCl for 1 h; (**D**) Progressive deletions of PG4s from the 5′-flanking regions in the *CLIC4* promoter were generated in the pSEAP2-Basic reporter plasmid. A375 cells were transfected with each reporter plasmid for 48 h. Media were collected and subjected to SEAP activity measurements. Data were expressed as means ± SD of three replicates. ### *p* < 0.001, as compared to p(−518, +285). *** *p* < 0.001 as compared to the adjacent promoter region. ns: non-significant difference.

To further elucidate the formation of the G4 structure in the *CLIC4* promoter region, we examined three G-rich sequences with a higher potential to form G4 structures, and named them as putative G-quadruplex-1, -2, and -3 (PG4-1, -2 and -3), as shown in Figure 2A. PG4-1 and PG4-2 are located on −396 to −364, and −352 to −322 of the sense strand, respectively; PG4-3 is on +50 to +79 of the antisense strand. The location of each PG4 within the *CLIC4* promoter is presented in Figure 1B. Circular dichroism (CD) spectra and one-dimensional (1D) imino-proton nuclear magnetic resonance spectroscopy (NMR) experiments were conducted to examine whether the three PG4s were involved in DNA secondary structure formation. CD bands at ~265 and ~290 nm characterize the signature of G4 DNA. Different spectra represent different conformation of G4. As shown in Figure 2B, both absorption bands appeared in PG4-1 and PG4-2, and the only band near 295 nm was increased in PG4-3 by treatment of K^+^ to induce G4 formation, implying the formation of G4 structure in all of these three PG4s. Furthermore, in NMR spectra (Figure 2C), multiple imino proton signals at 10.5~12 ppm were also found in all of these three PG4s in the presence of K^+^, confirming the formation of Hoogsteen hydrogen bonding for the quadruplex structure. In addition, there were also signals shown near 13 ppm representing typical Watson-Crick hydrogen bonding, suggesting the existence of the hairpin structure in PG4-1 and PG4-3. These results indicated the existence of DNA secondary structures in the *CLIC4* promoter region.

To examine whether PG4s in the promoter region play an important role in regulating *CLIC4* transcription, we constructed SEAP reporter plasmids containing different lengths of the *CLIC4* promoter that excluded PG4 at a point from upstream of the promoter, to elucidate the biological significance of each PG4. As shown in Figure 2D, when the first and second PG4 (PG4-1 and PG4-2) were removed, there was no significant change in the SEAP activity, suggesting that the sequences or the structures of PG4-1 and PG4-2 were not critical for *CLIC4* transcription. The deletion of −321 to −125 resulted in a decreased of SEAP activity implying the positive regulatory site lies in −518 to −125 observed in Figure 1A had been narrowed down to this region. Since the significance of PG4-1 and PG4-2 has been excluded, the following studies were focused on verifying the role of PG4-3 in regulating *CLIC4* transcription.

### 2.3. PG4-3 Is Involved in Regulating CLIC4 Transcription

**Figure 3:**
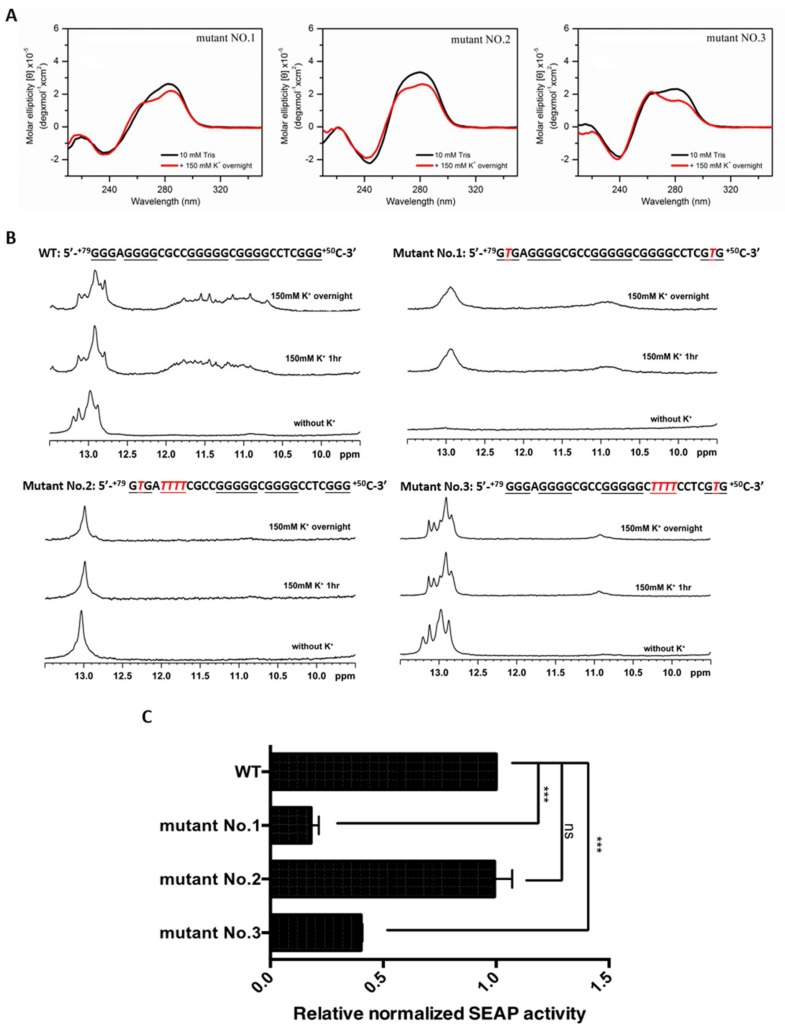
(**A**) The CD spectra of three mutants that disrupt the PG4-3 structure in Tris-HCl buffer without (black line) and with (red line) 150 mM KCl for overnight; (**B**) The imino proton NMR spectra of PG4-3 wild type (WT) and three mutants in Tris-HCl buffer without (**bottom** panel) or with 150 mM KCl for 1 h (**middle** panel) and overnight (**top** panel). G-tracts are underlined, and the mutation sites are marked in red italicized letters; (**C**) The effects of the mutations that disrupt the G4-3 structure. Each mutant of *CLIC4* p(−125, +285) was transfected in A375 cells. After 72 h, the media were collected for a SEAP assay. Data were expressed as the means ± SD of three replicates. *** *p* < 0.001, ns: non-significant difference as compared to WT control.

It has been shown that G4s in the promoter region could play a regulatory role for transcription in both positive and negative ways [27]. To elucidate the functional role of PG4-3 in regulating *CLIC4* transcription, we substituted G with T to disrupt the quadruplex structure. In the PG4-3 region, there are 29 bases (+79 to +50) comprising five G-tracts in the antisense strand of the *CLIC4* promoter. Three mutants were designed to disrupt the quadruplex formation. Mutant No. 1 was generated by changing 78G and 52G in the first and last G-tracts of the PG4-3 sequence to 78T and 52T, respectively. Mutant No. 1 was unable to form G4 as revealed by CD spectra with the absence of ~265 nm or ~290 nm bands in 150 mM K^+^ solution (Figure 3A). The NMR signals of PG4-3 at 10.5–12 ppm were diminished in Mutant No. 1 (Figure 3B). Meanwhile, the transcription activity of the reporter construct containing Mutant No. 1 was mostly decreased (Figure 3C), indicating that PG4-3 did play an important role in regulating CLIC4 expression. To further verify the G4 structure of the PG4-3 in gene transcription, we further constructed another two PG4-3 mutants. Except for the replacement of 78G with 78T in the first G-tract, the second G-tract (+75 to +72) was replaced with T in Mutant No. 2. On the contrary, Mutant No. 3 was designed by replace G with T in the penultimate G-tracts (+61 to +58), and 52G in the last G-tract. As shown in Figure 3AB, CD and NMR signals of G4 were also negligible in these two mutants. In the reporter assay (Figure 3C), the transcription activity in Mutant No. 3 was decreased; however, there was no significant change of transcription activity in Mutant No. 2.

**Figure 4:**
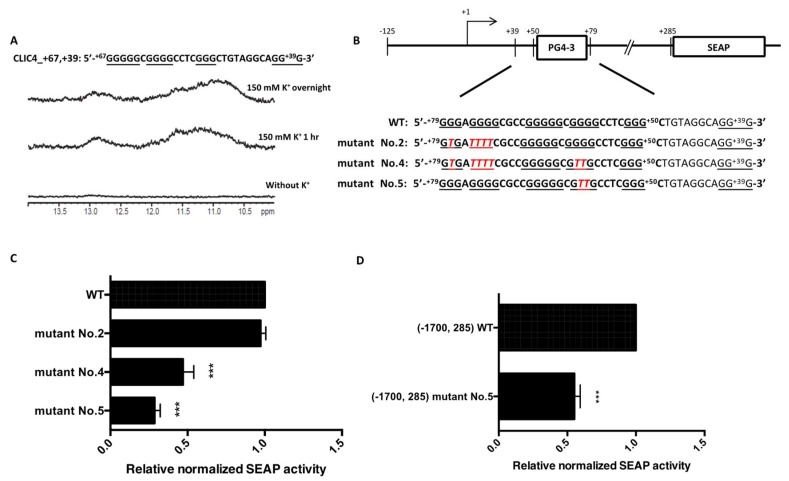
Further disruption of the remaining possible G4 structure formed in the Mutant No. 2 reporter plasmid. (**A**) NMR analysis of *CLIC4* +67 to +39, the possible G4-forming sequence in Mutant No. 2 in Tris-HCl buffer and 150 mM KCl, for 1 h and overnight; (**B**) Sequence of WT and mutants derived from Mutant No. 2—Mutants No. 4 and No. 5 contain the following G-tract at the 3′ end. The original PG4-3-forming region is shown in bold letters, G-tracts are underlined, and the mutated sequences in *CLIC4* p(−125, +285) plasmids are marked in red italicized letters; (**C**) SEAP activity of *CLIC4* p(−125, +285) mutants further disrupting the G4 forming motif in Mutant No. 2 were determined in A375 cells after transfection for 72 h. Data were expressed as the means ± SD of three replicates. *** *p* < 0.001 as compared to the WT. (**D**) SEAP activity of *CLIC4* p(−1700, +285) mutant No. 5 in A375 cells after transfection for 72 h. Data were expressed as means ± SD of three replicates. *** *p* < 0.001 as compared to *CLIC4* p(−1700, +285) WT.

Despite the disappearance of the G4 NMR signal in the synthetic Mutant No. 2 oligonucleotide, the reporter construct containing Mutant No. 2 sequence still exhibited normal transcription activity. We therefore further examined this contradictory result between the reporter construct and the synthetic nucleotide. Except for the five G-tracts in PG4-3, we found another G-tract at +39 to +41 immediately adjacent to PG4-3. We argued that this additional G-tract might be incorporated with the remaining three G-tracts, and it may have participated in forming another G4 structure in the reporter construct when the first two G-tracts were mutated in Mutant No. 2. To address the possibility that the Mutant No. 2 reporter construct still contained a G4-forming sequence, the sequence from +67 to +39, which included three remaining G-tracts and the additional G-tract, was synthesized and analyzed by NMR. As shown in Figure 4A, there were appreciable signals of a quadruplex structure at the 10.5~12 ppm region, suggesting that G4 formation occurs in the sequence from +67 to +39. Therefore, we further constructed different reporter plasmids containing other mutation sites (Figure 4B). Mutant No. 4 harbors extra mutation sites: 58G, 59G to 58T, 59T respectively, compared to Mutant No. 2. Meanwhile, Mutant No. 5 was created by only mutating 58G, 59G to 58T, 59T, respectively. As shown in Figure 4C, the reporter activity was again decreased once the remaining G4-forming sequences were mutated. Furthermore, in order to understand whether different promoter lengths would affect G4 formation in the plasmid, we also compared the transcription activity in a longer promoter region. A mutation site for Mutant No. 5 was inserted in a reporter plasmid with the longest promoter *CLIC4* p(−1700, +285) that we had constructed. As shown in Figure 4D, a significant decrease of transcription activity was found in Mutant No. 5.

**Figure 5:**
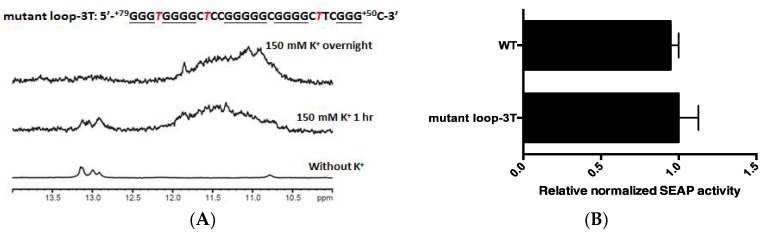
Strengthening of the PG4-3 structure. (**A**) The imino proton NMR spectra of the mutant *CLIC4* loop-3T that did not affect the G4-forming motif. G-tracts are underlined and the mutation sites are marked in red italicized letters; (**B**) The SEAP activity in A375 was determined after 72 h of transfection of *CLIC4* p(−125, +285) WT and the mutant loop-3T reporter plasmid. Data are expressed as the means ± SD of three replicates.

To further examine the importance of the PG4-3 G4 structure in regulating *CLIC4* transcription, we designed another mutant that we named loop-3T, in which only the non-G-tract sites: 76A, 70G, and 56C in the loops were mutated, respectively, to 76T, 70T, and 55T, which would not destroy G4-forming elements. The NMR result in Figure 5A showed that the G4 signals were retained in the mutant loop-3T. The reporter activity was also similar to the PG4-3 wild-type (WT) control (Figure 5B). These results indicate that the PG4-3 G4 structure within the promoter region plays an important role in regulating *CLIC4* transcription.

To elucidate the role of PG4-3 in regulating *CLIC4* expression, we used software to predict the possible candidate which could binds to the nucleotide sequences of PG4-3. SP1 and MAZ are the two candidate genes predicted to bind on this sequence and also have been reported to bind on G4 structure [29,30]. However, we found that knockdown SP1 or MAZ did not affect *CLIC4* mRNA expression nor transcription activity (Figure S1), indicating these TFs were not the key proteins affecting *CLIC4* transcription.

### 2.4. PG4-3 Acts as a Regulatory Element in the *CLIC4* Promoter Region of the Cell Chromosome

**Figure 6:**
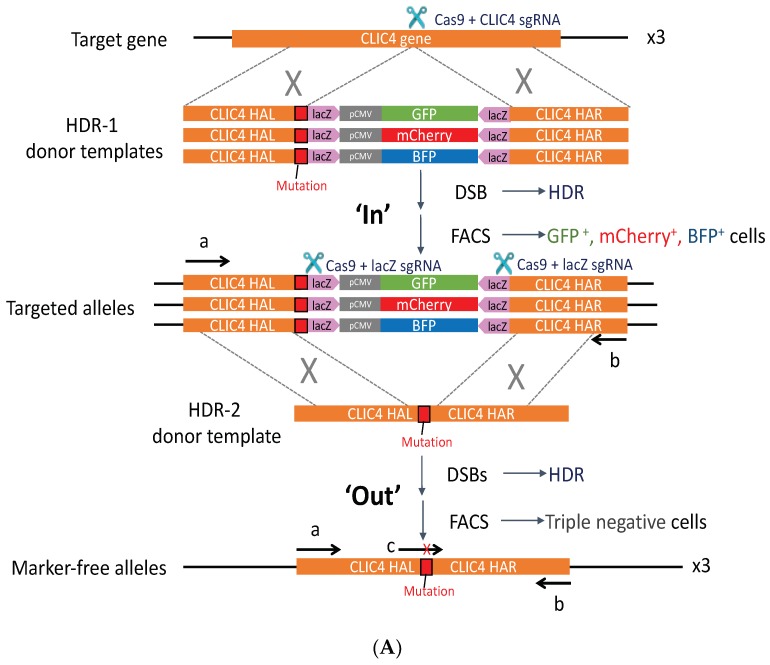

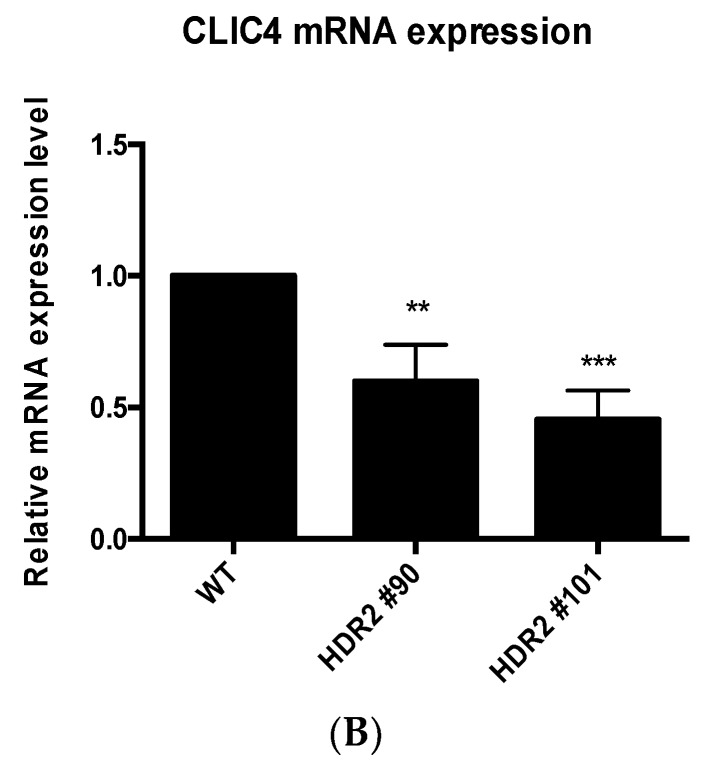
Mutation of the PG4-3 sequence at the endogenous *CLIC4* promoter. (**A**) Schematic diagram of the pop-in (‘In’) and pop-out (‘Out’) steps taken to modify PG4-3 to Mutant No. 3 in the *CLIC4* promoter. Briefly, in the pop-in step, CRISPR-Cas9 targeting by *CLIC4* sgRNA occurred next to the mutated site, and the HDR-1 donor templates consisting of a 1 kb sequence of *CLIC4* homology arms left and right (HAL and HAR, respectively), was interrupted by a fluorescence cassette: green fluorescent protein (*GFP*), *mCherry*, or blue fluorescent protein *(BFP*) gene driven by a CMV promoter and tagged with a lacZ sequence at both ends were introduced into the A375 cells. The lacZ sequence does not exist in the human genome and was later used in the pop-out step. Fluorescence-activated cell sorting (FACS) was used to isolate cells co-expressing the three fluorescence markers; primers ‘a’ and ‘b’ were used to check the genomic DNA size. In the pop-out step, the fluorescence markers were excised by two double strand breaks (DSBs) at the lacZ sites. The DSBs were repaired using the HDR-2 donor template. Loss of fluorescence expression in the cells (triple negative cells) were isolated. A TaqMan probe ‘c’, specifically purposed for recognizing the Mutant No. 3 sequence, was used to select clones for further sequencing of the PCR products that were generated with primers ‘a’ and ‘b’. Cleavage points are indicated by scissors; grey X represents homologous recombination; mutation site of Mutant No. 3 is shown as red box; arrow of a, b and c indicates the direction of synthesis; (**B**) *CLIC4* messenger RNA (mRNA) expression level in A375 with the genome edited to the Mutant No. 3 sequence. Total RNA was extracted for RT-PCR and real-time qPCR to analyze the *CLIC4* mRNA expression level. Data are expressed as the means ± SD of three replicates. ** *p* < 0.01, *** *p* < 0.001 as compared to A375 WT cells.

In order to verify whether this G4 structure actually has a regulatory function in the cell chromosome, we managed to disrupt the PG4-3 sequence in the *CLIC4* gene of the cell genome by using the CRISPR/Cas9 system. *CLIC4* is located on chromosome 1. The A375 cell line that was used in this study is hypotriploid, with three copies of chromosome 1 where the *CLIC4* gene is located. With regard to this fact, the two steps of editing: the pop-in/pop-out strategy developed by Cech and colleagues [31] was adopted with some modifications to assure precise editing by replacing the wild-type sequence (+79 to +50) with the Mutant No. 3 sequence in each chromosome. The workflow is illustrated in Figure 6A. In the pop-in step, the fluorescence markers were integrated into the cell genome by homology-directed repair (HDR). One of cell clones that co-expressed the three fluorescence markers with the expected genome size was then subjected to the pop-out step, in which the fluorescence markers were excised out and specifically repaired into the Mutant No. 3 sequence in cells that underwent HDR; therefore, triple-negative cells were isolated. In the end, two cell clones, HDR2 #90 and HDR2 #101, carrying PG4-3 Mutant No. 3 in the endogenous *CLIC4* promoter region, were generated. The representative sequencing results for *CLIC4* promoter region covering PG4-3 site of each cell clone could be found in supplementary folder (HDR2 single cell clones sequencing data). A lower level of endogenous mRNA expression was found in genome-edited cell clones carrying Mutant No. 3 that disrupted the PG4-3 structure (Figure 6B), indicated that PG4-3 in the promoter region of cell chromosome is important for *CLIC4* transcription.

## 3. Discussion

Several transcriptional factors in regulating *CLIC4* expression have been reported. Comparing the binding sites relative to the putative TSS in previous studies [13,14,15] with the known *CLIC4* mRNA TSS in the NCBI Reference Sequence, NM_013943.2, we found about 500 bp in differences. The promoter region from −500 to the TSS, and even to the translation start site of *CLIC4* have, in fact, not been well studied. This region is full of GC-rich sequences, implying the possible existence of DNA secondary structures. In this study, we demonstrated that the putative G-quadruplexes could be found in this high-GC-content region, and we further showed that this G4 structure did play an important role in the *CLIC4* promoter. Accordingly, we speculated that this secondary structure might cause past difficulties in amplifying and sequencing this promoter region, which might have impeded recent progress with studying the transcription mechanisms of *CLIC4*.

To investigate the effect of the G4 structure on the promoter with regard to transcription, studies have most commonly performed plasmid-based reporter assays. In this study, we first used a reporter assay to determine the significance of the PG4-3 structure in the *CLIC4* promoter, for its transcription. In addition, when the mutation sites that disrupted the G4 structure were placed in longer *CLIC4* promoter sequences within the reporter plasmid, this revealed similar results. Furthermore, it is known that chromatin status affects G4 formation [32]. Although the plasmid that was transfected into eukaryotic cells could be packed by histones and other proteins to become a nucleosome-like structure [33], it is not known whether it retained a similar status as a cellular chromosome, which may have had an impact on G4 formation. To this end, we then directly edited the promoter sequence in the cell chromosome by the CRISPR/Cas9 system, and we provide in vivo evidence to support the positive regulatory effect of PG4-3 on *CLIC4* transcription.

PG4-3 was on the anti-sense strand, which served as the template for RNA polymerase (RNAP)-mediated transcription. With regard to this, the formation of G4 could be the obstacle for RNAP, and the disruption of the G4 structure would result in the upregulation of transcriptional activity. However, as shown in Figure 3, we found the opposite phenomenon, with disruption of the G4-forming sequence leading to a decrease or no significant change in transcriptional activity. Therefore, we hypothesized that this PG4 might provide a binding site for protein(s) that favor *CLIC4* transcription or that prevent the binding of the repressor. SP1 and MAZ were two candidates that have been tested. However, we found that these two factors did not play the substantial role in regulating *CLIC4* expression. Meanwhile, in our preliminary EMSA studies, some unidentified proteins had increased binding onto the probes of Mutants 1 and 3 than that of the wild-type, suggesting the possible binding of repressors at the PG4-3 region.

There are five G-tracts in the originally identified PG4-3 sequence. In Mutant No. 2, we noticed that the additional G-tract might incorporate in the G4 formation when the first two G-tracts were mutated. The G4-forming sequence with a fifth G-tract has been observed in many oncogene promoters, and the ‘spare tires’ hypothesis was proposed [34]. For instance, guanine in G4 is susceptible to oxidation and has been shown to affect G4 stability [35]. The G4 in the vascular endothelial growth factor (*VEGF*) and endonuclease III-like protein 1 (*NTHL1*) promoters were modifiable by 8-oxo-7,8-dihydroguanine (OG) [34,36], and this caused the instability of the original G4 structure that was formed by the first four 4 G-tracts. After that, the fifth G-tract was able to act as a spare tire to maintain the G4 fold, and to allow the repair of DNA damage. Coincidentally, *CLIC4* is also a gene that can be regulated by reactive oxygen species (ROS)-induced oxidative stress. PDT-induced ROS downregulated *CLIC4* transcription [37], while TGF-β-induced ROS upregulated *CLIC4* transcription [38]. Whether OG modification take place on PG4-3 and affects G4 stability such that that the other two additional G-tracts can take part in G4 formation, deserves further investigation.

## 4. Materials and Methods

### 4.1. Cell Culturing

Human melanoma A375 cells (American Type Culture Collection, Manassas, VA, USA) were cultured in Dulbecco’s modified Eagle’s medium (DMEM) supplemented with 10% fetal bovine serum (FBS), and grown at 37 °C under 5% CO_2_.

### 4.2. Circular Dichroism (CD) Spectroscopy

CD experiments were conducted using a spectropolarimeter (J-815, Jasco, Tokyo, Japan) with a bandwidth of 2 nm at a scan speed of 50 nm/min and a step resolution of 0.2 nm over the spectral range of 210–350 nm. The DNA sample concentrations were 4 μM in 10 mM Tris-HCl (pH 7.5), and a stock solution of 3 M KCl (Sigma-Aldrich, St. Louis, MO, USA) was added to the DNA samples to reach a final K^+^ concentration of 150 mM. The observed signals were baseline subtracted.

### 4.3. NMR

The unlabeled oligonuleotides synthesized by Bio Basic (Markham, ON, Canada) were prepared to 100 μM in 10 mM Tris-HCl (pH 7.5) with or without 150 mM KCl, followed by denaturing at 95 °C for 5 min and slowly annealed to 25 °C. The strand concentrations of the NMR samples were 100 μM containing 10% D_2_O in 10 mM Tris-HCl (pH 7.5) or 150 mM K^+^ conditions with an internal reference of 0.01 mM DSS (4,4-dimethyl-4-silapentane-1-sulfonic acid), and they were analyzed by Bruker AVIII (Billerica, MA, USA) 500 MHz spectrometers equipped with a prodigy probehead, and on a Bruker AVIII 800 MHz NMR spectrometer equipped with a cryoprobe at 25 °C. 1D imino proton NMR spectra were recorded using a WATERGATE for water suppression.

### 4.4. Reporter Assay

The *CLIC4* sequence from −1700 to 285 relative to the transcription start site (+1) was generated from genomic DNA of A375 cells, and cloned into the pSEAP2-Basic vector (Clontech, Mountain View, CA, USA). After transfection in A375 cells with TurboFect (Thermo Scientific™, Waltham, MA, USA) for 48 to 72 h, the culture medium was collected and analyzed for SEAP activity by measurement of the hydrolysis of p-nitrophenyl phosphate (pNpp) with a spectrophometer at OD_405_. MTT assay was used for the normalization of cell numbers.

### 4.5. CRISPR/Cas9

*CLIC4* sgRNA targeting near *CLIC4* PG4-3 was selected and synthesized by in vitro transcription. HDR-1 donor templates containing different fluorescence cassettes: *GFP*, mCherry, and *BFP*, driven by a CMV promoter with a lacZ sequence on both ends (kindly provided by Dr. Steve Lin) flanking a 1 kb sequence of *CLIC4* homology arms upstream and downstream of the PG4-3 Mutant No. 3 mutation site were generated by Gibson assembly. Cas9, *CLIC4* sgRNA, and three fluorescence donor templates were introduced to A375 cells by nucleofection with the 4D-Nucleofector^TM^ system and SF kit (Lonza, Basel, Switzerland) under the FF-120 program. Single cells co-expressing three fluorescence markers were isolated by FACS for clonal expansion, and the genomic sizes of the sequences containing the fluorescence cassettes were confirmed by PCR. In the pop-out step, Cas9, sgRNA targeting the lacZ sites, and the HDR-2 donor template only containing the *CLIC4* homology arms with the mutation sites, were again nucleofected in the pop-in cell clone. Single cells without fluorescence were sorted by FACS. The genomic DNA edited into the Mutant No. 3 sequence was analyzed by PCR and Custom TaqMan^®^ Gene Expression Assays, SM ID: APFVMGD, which was designed to specifically anneal to the *CLIC4* mutation site. Cell clones harboring the mutant sequence were further confirmed by sequencing.

### 4.6. Real-Time PCR Analysis

Total RNA was extracted using TRIzol reagent (Invitrogen, Carlsbad, CA, USA) following the manufacturer’s instructions accordingly. A total of 1 µg RNA was used to synthesize complementary DNA (cDNA) by reverse transcription. The cDNA product was used as a template for real-time PCR analysis using the ABI Fast SYBR^®^ Green Master Mix Kit (Thermo Fisher Scientific, Waltham, MA, USA) with the ABI StepOne system (Thermo Fisher Scientific). The primer sequences were as follows: CLIC4 (sense), 5′-GCAGTGATGGTGAAAGCATAG-3′; CLIC4 (anti-sense), 5′-TATAAATGGTGGGTGGGTCC-3′; GAPDH (sense), 5′-GACCACAGTCCATGCCATCA-3′; GAPDH (anti-sense), 5′-GTCCACCACCCTGTTGCTGTA-3′.

### 4.7. Statistical Analysis

All results were obtained from three independent experiments, and each value was expressed as the mean ± SD. The two-tailed Student’s *t*-test was used to compare the differences between pairs of means. *p* < 0.05 was considered significant.

## 5. Conclusions

The G4 structure formed by PG4-3 in the *CLIC4* promoter region may act as a regulatory element in regulating *CLIC4* gene transcription, as shown in the reporter assay, as well as in the CRISPR-modified A375 cell clone with mutated PG4-3.

## Supplementary Materials

Supplementary materials can be found at http://www.mdpi.com/1422-0067/19/9/2678/s1. Figure S1: Regulation of MAZ and SP1. Supplementary Folder: HDR2 single cell clones sequencing data. The representative sequencing results of HDR2 #90 and HDR2 #101 for CLIC4 promoter region covering PG4-3 site.
