# Supplementary material for: A G-Quadruplex Structure in the Promoter Region of CLIC4 Functions as a Regulatory Element for Gene Expression
Source: Int J Mol Sci. 2018 Sep 10;19(9):2678. doi: 10.3390/ijms19092678 (PMC6165315; doi:10.3390/ijms19092678)
Supplement: Supplementary file 1 [file ijms-19-02678-s001.zip › Supplementary_/supplementary.docx]

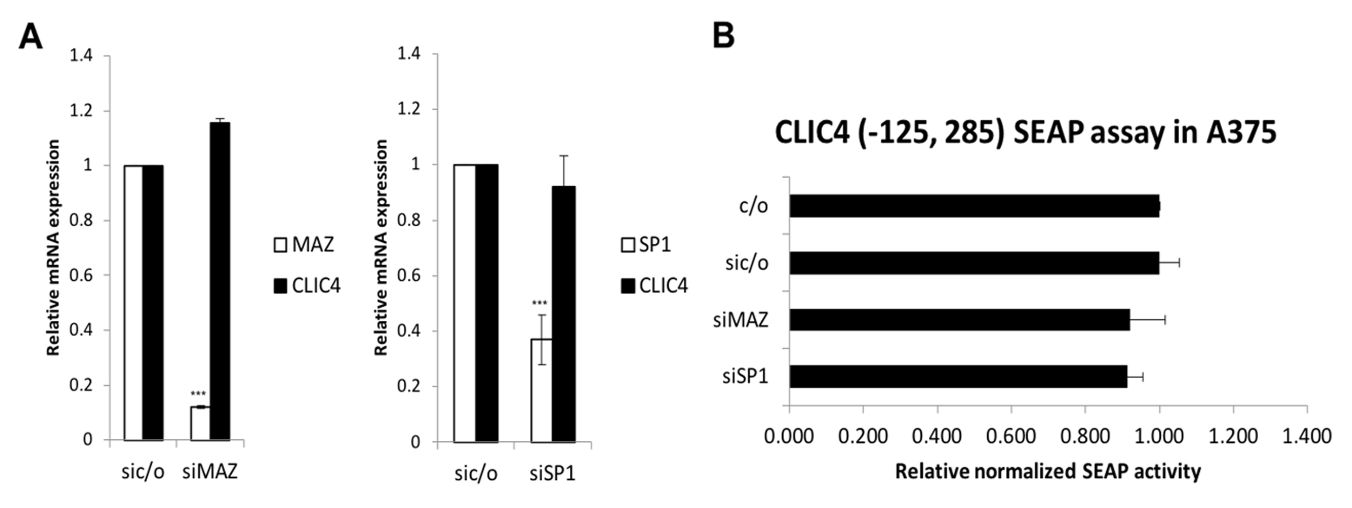


**Figure S1.** Regulation of MAZ and SP1. (A) Small interfering RNA (siRNA) of MAZ or SP1 was transfected in A375 cells for 48 h. MAZ or SP1, and *CLIC4* mRNA were analyzed by RT-PCR. (B) *CLIC4* p(−125, 285) reporter plasmid and siRNA of MAZ or SP1 were co-transfected in A375. After 48 h, the media was collected for the SEAP assay.
